# A sibling species of *Platythyrea clypeata* Forel, 1911 in southeast Asia (Hymenoptera, Formicidae, Ponerinae)

**DOI:** 10.3897/zookeys.729.21378

**Published:** 2018-01-16

**Authors:** Natthaporn Phengsi, Weeyawat Jaitrong, Jiraporn Ruangsittichai, Salinee Khachonpisitsak

**Affiliations:** 1 Biological Science Program, Department of Biology, Faculty of Science, Burapha University, 169 Long-Hard Bangsaen Road, Sanesuk, Mueang, Chon Buri, 20131 Thailand; 2 Natural History Museum, National Science Museum, Technopolis, Khlong 5, Khlong Luang, Pathum Thani, 12120 Thailand; 3 Department of Medical Entomology, Faculty of Tropical Medicine, Mahidol University, 420/6 Ratchawithi Road, Ratchathewi, Bangkok, 10400 Thailand; 4 Department of Biology, Faculty of Science, Burapha University, 169 Long-Hard Bangsaen Road, Sanesuk, Mueang, Chon Buri, 20131 Thailand

**Keywords:** new species, taxonomy, Thailand, *Platythyrea*, Ponerinae

## Abstract

A new species of the rarely collected ant genus *Platythyrea* Roger, 1863 closely related to *Platythyrea clypeata* Forel, 1911 is described and illustrated based on the worker caste under the name *Platythyrea janyai* **sp. n.** This species is distributed in southern Thailand and western Malaysia, while *P. clypeata* is distributed in Sri Lanka, Vietnam, Laos, and Thailand in the areas north of the Isthmus of Kra. *Platythyrea clypeata* is newly recorded from Thailand from dead wood on the forest floor. The type series of *P. janyai* was also collected from rotten wood on the forest floor.

## Introduction

*Platythyrea* Roger, 1863 is a ponerine genus of the tribe Platythyreini, with *Pachycondyla punctata* Bingham, 1903 as the type species (Bolton 2003, Schmidt and Shattuck 2014). The genus is mainly pantropical in distribution, with some species also occurring in subtropical regions of the New World, Africa, Asia, and Australia (Bolton 2003, Schmidt and Shattuck 2014, Antweb 2017). Members of the genus are reported to nest in hollow branches or other preformed cavities in live or fallen trees, and to forage on tree trunks or other vegetation (Brown 1975, Ito 1994, 2016, Djiéto-Lordon et al. 2001, Molet and Peeters 2006, Yéo et al. 2006). Some large African species nest at the base of termite mound or under rocks (Brown 1975, Schmidt and Shattuck 2014).

At present, 38 extant species have been described within the genus with 9, 17, 6, and 8 species from the Neotropical, Ethiopian, Australian and Oriental regions, respectively (Schmidt and Shattuck 2014, Antweb 2017). Six species have been recorded in southeast Asia (*Platythyrea bidentata* Brown, 1975; *P. clypeata* Forel, 1911; *P. inermis* Forel, 1910; *P. parallela* (F. Smith, 1859); *P. quadridenta* Donisthorpe, 1941; and *P. tricuspidata* Emery, 1900). Jaitrong and Nabhitabhata (2005) recorded only three species, *Platythyrea parallela*, *P. quadridenta*, and *P. tricuspidata* from Thailand. A recent examination of *Platythyrea* specimens from Laos, Thailand, and western Malaysia recognised an additional two closely related species from these areas; one is new to science and is described herein, and the other (*P. clypeata*) is new to Thailand and is redescribed based on the worker and dealate queen.

## Materials and methods

Holotype and paratypes of the new species are point-mounted and were examined along with other specimens of *Platythyrea* deposited in the Ant Museum, Faculty of Forestry, Kasetsart University, Bangkok, Thailand and the Natural History Museum of the National Science Museum, Thailand. Two dealate queens of *P. clypeata* collected from Laos were compared with the high resolution images of the *P. clypeata* holotype (alate queen) available on Antweb (2017). The holotype, paratype, and non-type workers of the new species were compared with workers from the colony from Laos to which the dealate queen belonged.

Most morphological observations were made with a ZEISS Discovery.V12 stereomicroscope. Multi-focused montage images were produced using NIS element 3.7 from a series of source images taken by a Nikon MNB42100 digital camera attached to a Nikon ECLIPSE E600 microscope. The holotype and paratypes were measured using a micrometre. All measurements are expressed in millimetres to the hundredths place.

Abbreviations used for the measurements and indices are as follows:

**TL** Total length in profile, roughly measured from the anterior margin of the head to the tip of the gaster in outstretched specimens.

**HL** Maximum head length in full-face view, measured from the anterior clypeal margin to the midpoint of a line drawn across the posterior margin of the head.

**HW** Maximum head width in full-face view, measured just behind the eyes.

**SL** Scape length excluding the basal constriction and condylar bulb.

**EL** Eye length, the maximum length of the eye in profile.

**WL** Weber’s length, the diagonal length of the mesosoma in profile from the anterior margin of the pronotum to the posteroventral angle of the metapleuron, excluding the neck.

**PL** Petiole length measured from the anterior margin of the peduncle to the posterior-most point of the tergite in profile.

**PH** Petiole height, the maximum height of the petiole in profile view.

**PW** Petiole width, the maximum width of the petiole in dorsal view.

**CI** Cephalic index. HW/HL × 100.

**EI** Eye index. EL/HW × 100.

**SI** Scape index. SL/HW × 100.

Abbreviations of the type depositories and others are as follows:

**AMK** Ant Museum, Faculty of Forestry, Kasetsart University, Bangkok, Thailand

**THNHM** Natural History Museum of the National Science Museum, Pathum Thani, Thailand

**MHNG** Muséum d’Histoire naturelle de la Ville de Genève, Switzerland

The general terminology of the worker ants follows Hölldobler and Wilson (1990) and Bolton (1994). For the important characters of the worker in the genus *Platythyrea* used in this paper, see Brown (1975), Bolton (2003) and Schmidt and Shattuck (2014). Queen and male characters of the genus, see Brown (1975) and Yoshimura and Fisher (2007).

In addition to morphometric measurements, Scanning Electron Microscope images of *Platythyrea* were made at Microscopic Center, Faculty of Science, Burapha University with a LEO 1450 VP scanning electron microscope on gold coated specimens.

## Taxonomy

### Platythyrea janyai sp. n.

Taxon classificationAnimaliaHymenopteraFormicidae

http://zoobank.org/E162024F-7730-41E9-9C3F-4667A2C3ECDD

Figs 1 , 5B1–B3

#### Holotype.

Worker from Southern Thailand, Phatthalung Province, Si Banphot District, Riang Thong Waterfall, Khao Pu Khao Ya National Park, 28.IX.2007, W. Jaitrong leg., Colony no. WJT07-TH-2060 (THNHM-I-02392) deposited in THNHM.

#### Paratypes.

Three workers, same data as the holotype (THNHM-I-02393 to THNHM-I-02395).

#### Non-type material examined.

Two workers from Southern Thailand, Trang Province, Na Yong District, Khao Chong Botanical Garden, 7.XI.2014, W. Jaitrong leg., Colony No. WJT071114-2 (THNHM-I-02421 to THNHM-I-02422); one worker from western Malaysia, Selangor, Ulu Gombak, 22.III.2013, F. Ito leg. (THNHM-I-02465).

#### Measurements and indices.

***Holotype.*** TL 6.63 mm; HL 1.42 mm; HW 1.06 mm; SL 1.39 mm; EL 0.20 mm; WL 2.21 mm; PL 0.73 mm; PH 0.53 mm; PW 0.40 mm; CI 74, EI 18, SI 131. ***Paratypes*** (n = 3). TL 6.67-6.96 mm; HL 1.45 mm; HW 1.06 mm; SL 1.42 mm; EL 0.20 mm; WL 2.31 mm; PL 0.79 mm; PH 0.53 mm; PW 0.40 mm; CI 72, EI 18, SI 134.

**Figure 1.**
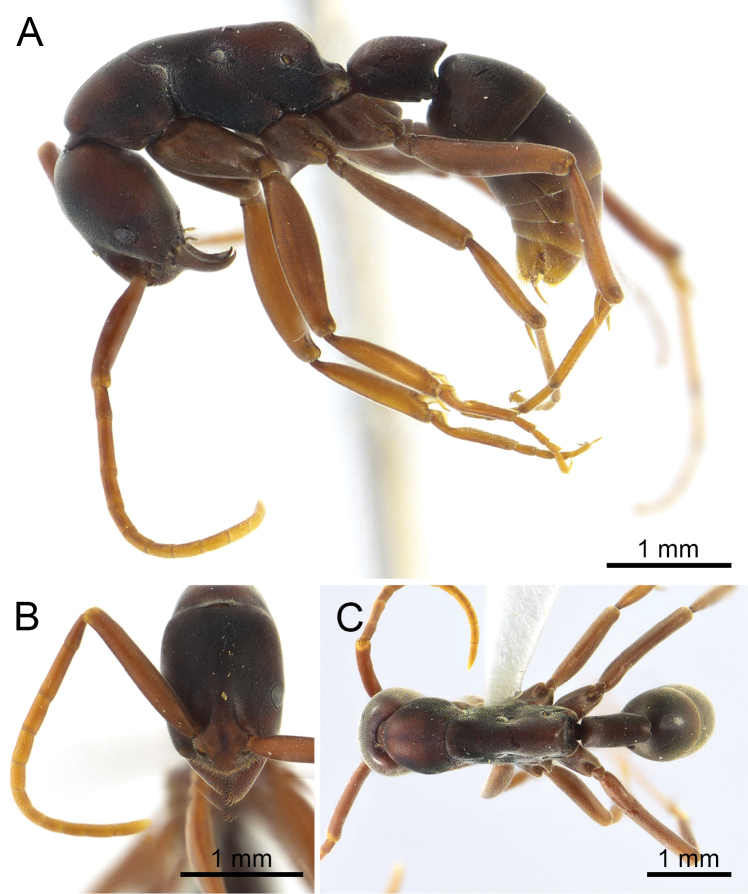
*Platythyrea janyai* sp. n. (holotype worker, THNHM-I-02392). **A** Body in profile view **B** Head in full-face view **C** Body in dorsal view.

#### Worker description.

*Head.* Head in full-face view subrectangular, clearly longer than broad, with sides weakly convex, occipital corner round, and posterior margin almost straight; antenna relatively long; scape slender, clearly extending beyond posterolateral corner of head; antennal segment II narrow, 1.6 times as long as segment III; III longer than each of segments IV–XII; clypeus broad, in profile with median portion distinctly convex, in full-face view lateral portion narrow and anterior margin clearly convex; mandible triangular, masticatory margin with a large apical tooth, followed by 9–10 smaller teeth, larger and smaller teeth alternating, but the series as a whole decreasing in size toward basal tooth; basal margin of mandible without denticle; eye slightly convex, located laterally anterior to mid-length of head, relatively large, 0.20 mm in maximum diameter, with eleven ommatidia on longest axis, distance between mandibular base and anterior margin of eye 1.5 times as long as maximum eye length; with head in profile, distance between posterior margin of eye and occipital corner of head 3.4 times as long as distance between mandibular base and anterior margin of eye; frontal lobes relatively close to each other, with roundly convex lateral margins; antennal socket horizontal, in plane of transverse axis of head, and in dorsal view, half concealed by frontal lobe.

*Mesosoma* elongate, in profile with weakly convex dorsal outline; promesonotal suture distinct; metanotal groove absent; mesopleuron not clearly demarcated from mesonotum, but can be separated from metapleuron by a shallow furrow; metapleuron not demarcated from lateral face of propodeum; propodeum with almost straight dorsal outline; propodeal junction rounded; declivity of propodeum shallowly concave; seen from back propodeal declivity tapering above; propodeal spiracle opening elliptical; legs very long.

*Petiole* cylindrical and sessile, clearly longer than high and broad, its dorsal outline almost straight; with petiole in profile posterodorsal corner with acute angles overhanging declivity of petiole; declivity of petiole shallowly concave; in dorsal view petiole rectangular, its posterior margin concave medially; subpetiolar process weakly developed, subtriangular, located anteroventrally; ventral outline of petiole weakly convex.

*Sculpture*. Head (including antennal scape), mesosoma, petiole and gaster finely and densely micropunctate; coxae and femora superficially reticulate but shiny.

*Pubescence* white, very short and fine, distributed over whole body and appendages, longer and more oblique on anterior clypeal margin, tip of mandible and hypopygium; setae absent.

*Colouration*. Dorsum of head dark brown, while lateral face of head reddish brown; mesosoma, petiole and gaster dark brown to reddish brown (tip of gaster yellowish); antenna and legs yellowish brown (funicular segments paler than scape).

#### Ecology.

The type series and all material examined of *P. janyai* were collected from small dead wood on the forest floor in lowland rainforests.

#### Etymology.

The specific name is dedicated to Mr Janya Jareanrattawong of the Royal Forest Department, Thailand who kindly helped W. Jaitrong in ant collecting in southern Thailand.

#### Distribution.

Southern Thailand (Phatthalung and Trang Provinces) and western Malaysia.

### Platythyrea clypeata

Taxon classificationAnimaliaHymenopteraFormicidae

Forel, 1911

Figs 2 , 3 , 5A1-A3

Platythyrea clypeata Forel, 1911: 378; Brown 1975: 50; Bolton 1995: 336; Xu and Zeng 2000: 214; Schmidt and Shattuck 2014: 51. Senior synonym of P. thwaitesi: Brown, 1975: 8.
Platythyrea thwaitesi Donisthorpe, 1931: 496. Junior synonym of P. clypeata: Brown 1975: 8.

#### Type.

The syntype alate queen from “Pays de Moïs”, Cochinchina française (S.E. Asia), deposited in MHNG (not examined).

#### Non-type material examined.

Nine workers, eastern Thailand, Chachoengsao Province, Tha Takiab District, Khao Ang Reu Nai Wildlife Sanctuary, 27.IX.2002, W. Jaitrong leg., Colony no. WJT270902-1 (THNHM-I-02423 to THNHM-I-02431); three workers, same locality, date and collector, Colony no. WJT270902-1 (THNHM-I-02432 to THNHM-I-02434); six workers, eastern Thailand, Sa Kaeo Province, Khao Ang Reu Nei Wildlife Sanctuary, 26.VI.2003, W. Jaitrong leg., Colony no. WJT03-TH-228 (THNHM-I-02435 to THNHM-I-02440); 23 workers and one male, eastern Thailand, Chanthaburi Province, Soi Dao District, 14.V.2008, W. Jaitrong leg., Colony no. WJT08-E065 (THNHM-I-02441 to THNHM-I-02453). Ten workers and one dealate queen, Laos, Vientiane, Pak Ngum District, Ban Phang Dang, ca. 300 m alt, 14.VI.2010, W. Jaitrong leg., Colony no. WJT-LAO-143 (THNHM); one dealate queen from same locality and collector, 12.VI.2010 (THNHM).

#### Measurements and indices.

TL 5.74–6.20 mm; HL 1.29–1.39 mm; HW 0.86–0.89 mm; SL 1.12–1.18 mm; EL 0.10 mm; WL 1.85–2.05 mm; PL 0.66–0.73 mm; PH 0.46–0.53 mm; PW 0.40–0.43 mm; CI 61–69, EI 11, SI 125–138.

**Figure 2.**
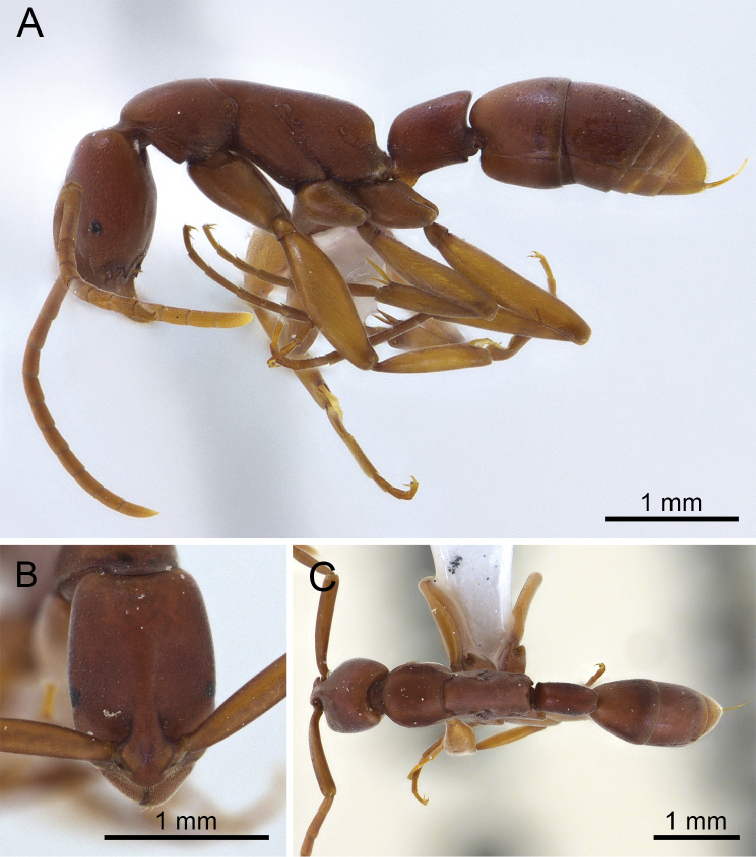
*Platythyrea clypeata* (non-type worker from Chanthaburi Province, THNHM-I-02445). **A** Body in profile view **B** Head in full-face view **C** Body in dorsal view.

#### Worker redescription.

*Head.* Head in full-face view rectangular, clearly longer than broad, with sides weakly convex or almost parallel, occipital corner roundly convex, and posterior margin feebly concave; antennal scape slender, relatively short, slightly extending beyond posterolateral corner of head (by 1/4 of its length); clypeus narrow, in profile with median portion distinctly convex, in full-face view lateral portion relatively broad and anterior margin clearly convex; mandible triangular, its masticatory margin with a large apical tooth, followed by ten smaller teeth (including basal tooth), large and smaller teeth alternating, but the series as a whole decreasing in size; basal margin of mandible without denticle; eye flat, located laterally at anterior to mid-length of head, very small, 0.10 mm in maximum diameter, with five ommatidia along longest axis; distance between mandibular base and anterior margin of eye approximately three times as long as maximum eye length; with head in lateral view, distance between posterior margin of eye and occipital corner of head 2.7 times as long as distance between mandibular base and anterior margin of eye; frontal lobes close to each other and rounded; frontal carinae strongly narrowed posteriorly.

*Mesosoma* elongate, in profile with almost straight dorsal outline; promesonotal suture distinct; mesopleuron demarcated from mesonotum and metapleuron by shallow furrows; propodeum in profile with almost straight dorsal outline; propodeal junction obtusely angulated; declivity of propodeum shallowly concave; seen from back propodeal declivity rounded above; propodeal spiracle opening elliptical; legs relatively long.

*Petiole* cylindrical and sessile, slightly longer than high and clearly longer than broad, its dorsal outline almost straight; in profile posterodorsal corner forming an acute angle; declivity deeply concave; in dorsal view node rectangular, slightly narrower posteriorly, its posterior margin convex and with shallow median concavity; subpetiolar process developed, located anteroventrally, subtriangular, its apex truncate and pointed forward; ventral outline of petiole feebly concave.

*Sculpture.* Dorsum of head finely punctate; lateral face of head behind, above and below eye punctate with dense foveae; dorsum of mesosoma with fine micropunctures similar to those on dorsum of head; lateral faces of pronotum, metapleuron and propodeum punctate with sparse shallow foveae; petiole finely micropunctate; gastral tergites I and II finely reticulate; antennal scape finely micropunctate; coxae microreticulate with smooth and shiny interspaces.

*Pubescence* white, very short and fine; setae present on tip of gaster.

*Colouration.* Head, mesosoma, petiole, and gaster reddish brown to dark brown (tip of gaster yellowish); antenna and legs yellowish brown to reddish brown (flagellum paler than scape).

**Figure 3.**
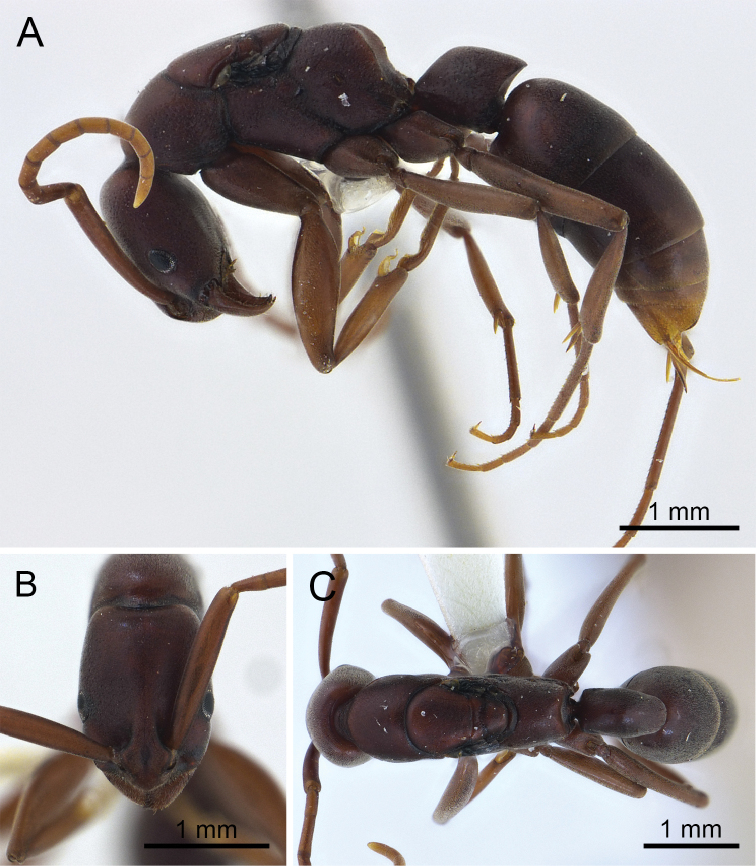
*Platythyrea clypeata* (queen from Laos, Colony no. WJT10-LAO143). **A** Body in profile view **B** Head in full-face view **C** Body in dorsal view.

#### Measurements and indices

(n = 2). TL 7.49 mm; HL 1.52 mm; HW 1.06 mm; SL 1.32 mm; EL 0.20 mm; WL 2.31 mm; PL 0.79 mm; PH 0.73 mm; PW 0.46 mm; CI 69, EI 18, SI 125.

#### Dealate queen description.

Body size slightly larger than worker. *Head*. Head in full-face view rectangular, clearly longer than broad with convex sides and almost straight posterior margin, occipital corner roundly convex; antennal scape extending beyond posterolateral corner of head by approximately 1/4 of its length; eye relatively large and convex, located anterior to mid-length of head, 0.20 mm in maximum diameter with ca. 17 ommatidia on the longest axis; frontal lobe and frontal carina similar to those in worker caste; distance between anterior margin of eye and mandibular base almost as long as eye length; ocelli clearly absent.

*Mesosoma* in profile with slightly convex dorsal outline; pronotum long and broad; mesoscutum trapezoidal, anterior edge clearly convex in dorsal view, separated from mesoscutellum by a shallow but wide suture and from pronotum by narrow suture; parapsidal lines indistinct, relatively long, straight and running anteriorly to mid-length of mesoscutum; mesoscutellum almost as long as broad; metanotum very short, separated from mesoscutellum and propodeum by deep grooves; propodeum relatively long; mesopleuron broad, anepisternum not demarcated from katepisternum; propodeal junction obtusely angulated; declivity of propodeum shallowly concave; seen from back propodeal declivity rounded above.

*Petiole* in profile view relatively short, rhombus, almost as long as high, its anterior margin weakly convex while posterior margin concave; declivity of petiole shallowly concave; subpetiolar process low and subtriangular, located anteroventrally, its apex pointed forward; ventral outline of petiole feebly concave. Gaster larger than in worker.

*Sculpture, colouration*, and *setae* similar to those of worker caste.

#### Distribution.

Sri Lanka, Vietnam, China (?) and Thailand (new record).

#### Ecology.

*Platythyrea clypeata* occurs in lowland (200–300 m alt) and inhabits primary and disturbed forests. All colonies of this species were collected from dead wood on the forest floor in an advanced stage of decomposition.

### Key to the southeast Asian species of genus *Platythyrea* based on the worker caste

**Table:** 

1	Frontal carinae very widely spaced, not continuing beyond level of posterior margin of antennal insertions (Fig. 7A); propodeal spiracle opening circular	**2**
–	Frontal carinae relatively narrowly separated, extending far beyond level of posterior margin of antennal insertions where space between them is very narrow (Fig. 7B); propodeal spiracle opening elliptical	**6**
2	In dorsal view, posterior margin of petiole with 2-3 distinct spines, teeth or blunt angles (Fig. 8A, B)	**3**
–	In dorsal view, posterior margin of petiole without distinct spines, teeth or sharp angles (Fig. 8C)	**5**
3	In dorsal view, posterior margin of petiole clearly concave with distinct lateral blunt angles; petiole almost as long as high	**4**
–	In dorsal view, posterior margin of petiole with 3 distinct spines; petiole longer than high (Fig. 8B)	***P. tricuspidata***
4	In profile view, propodeum armed with a pair of short teeth or tubercles (Fig. 9); lateral face of pronotum punctate with dense foveae	***P. quadridenta***
–	In profile view, propodeum unarmed; dorsum curving evenly into declivity (see fig. 28 in Brown 1975); lateral face of pronotum punctate with sparse foveae	***P. bidentata***
5	In dorsal view, petiole clearly longer than broad; antennal scape relatively short, not reaching posterolateral corner of head	***P. pararella***
–	In dorsal view, petiole almost as long as broad; antennal scape relatively long, slightly extending beyond posterolateral corner of head	***P. inermis***
6	Head relatively shorter (CI 72–74); eye clearly larger (EL 0.20 mm with 11 ommatidia on longest axis); eye convex; dorsum and lateral face of head finely micropunctate without foveae; in profile view petiole clearly longer than high and in dorsal view node of petiole anteriorly as broad as posteriorly; ventral outline of petiole weakly convex	***P. janyai* sp. n.**
–	Head relatively longer (CI 61–69); eye clearly smaller (EL 0.10 mm with 5 ommatidia on longest axis); eye flat; dorsum and lateral face of head finely punctate with dense shallow foveae; in profile view petiole slightly longer than high and in dorsal view node of petiole slightly narrower posteriorly; ventral outline of petiole feebly concave	***P. clypeata***

## Discussion

**Figure 4.**
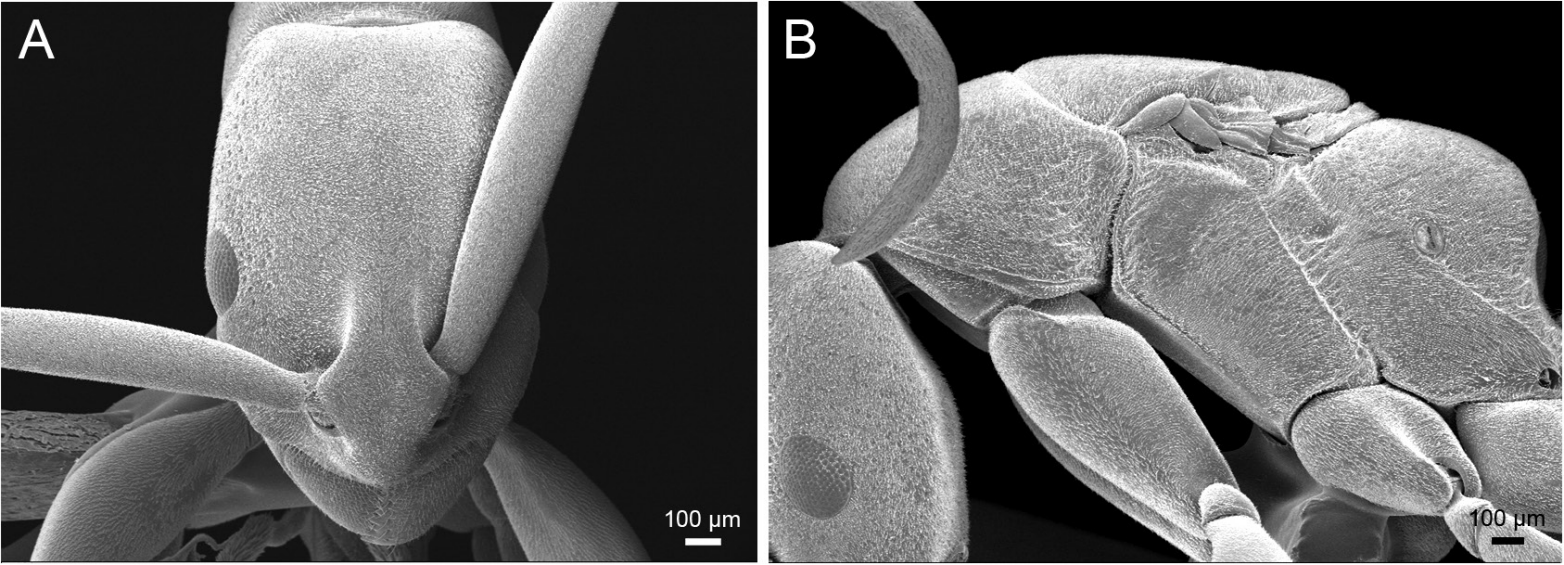
SEM images of *Platythyrea clypeata* (dealate queen from Laos, Colony no. WJT10-LAO143). **A** Head in full-face view **B** Mesosoma in profile view.

**Figure 5.**
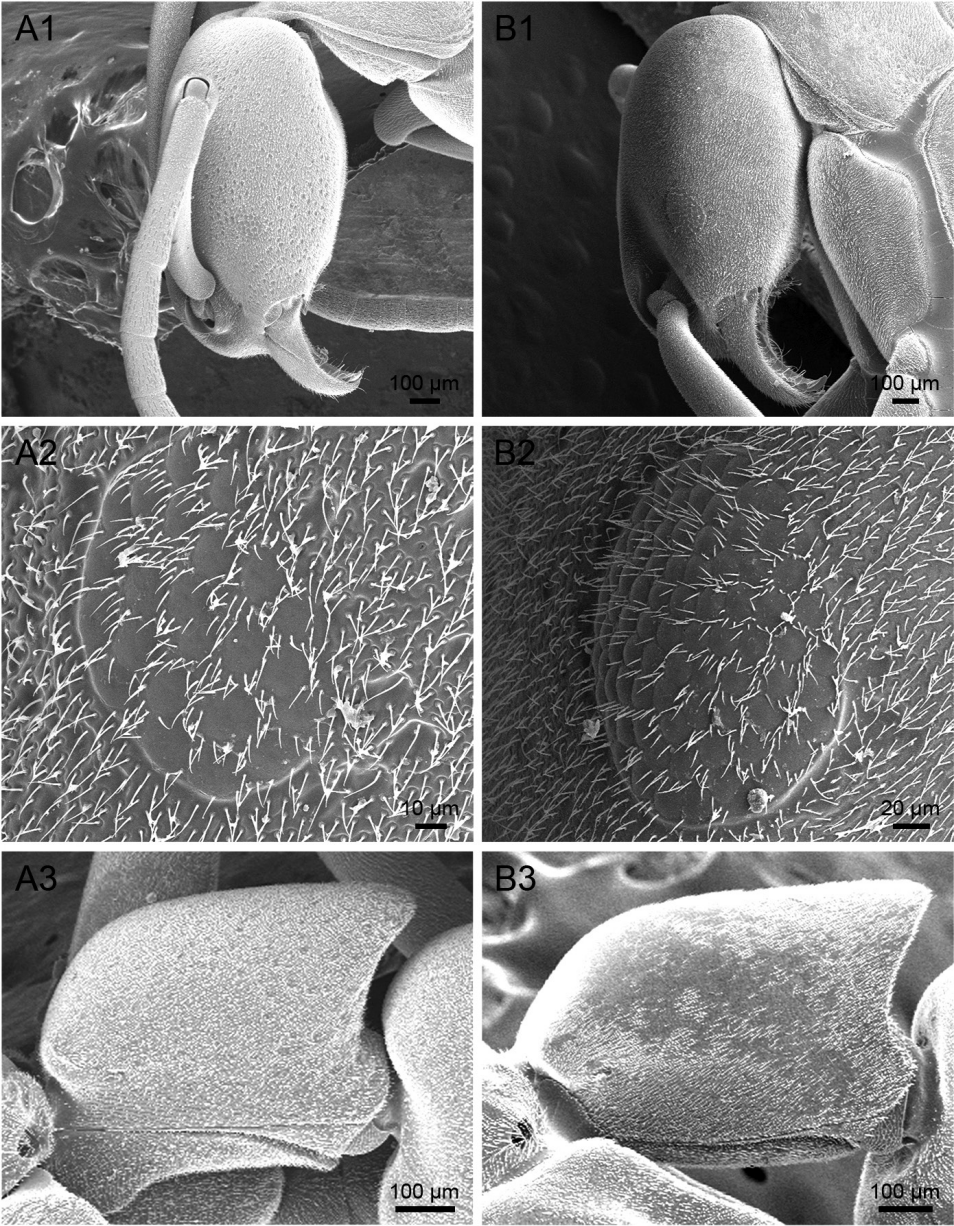
SEM images of *Platythyrea clypeata* (**A1–A3**) and *P. janyai* (**B1–B3**). **A1, B1** Sculpture on lateral face of head **A2, B2** Ommatidia of eye **A3, B3** Petiole in profile view.

Two dealate queens of the *P. clypeata* species group collected from Laos were compared with the high-resolution images of the *P. clypeata* holotype (alate queen from “Pays de Mois, Cochinchina française”) available on Antweb (2017). They have similar body sizes to those mentioned in Brown (1975: 50) and external morphological characteristics to the *P. clypeata* holotype. Additionally, Brown (1975) pointed out that alate queen of *P. clypeata* (holotype) completely lacks ocelli, which has also been observed in the Lao specimens (Fig. 4A). Workers of the *P. clypeata* species group collected from Eastern Thailand agree in most characters with the workers of a colony with a dealate queen (WJT-LAO-143) from Laos, while workers collected from Southern Thailand and western Malaysia differ from Laos and Eastern Thailand specimens. Thus, we here identify the specimens collected from Laos and Eastern Thailand as *P. clypeata* and those from Southern Thailand and western Malaysia as a new species, *P. janyai* sp. n. (see Fig. 5 for comparison).

**Figure 6.**
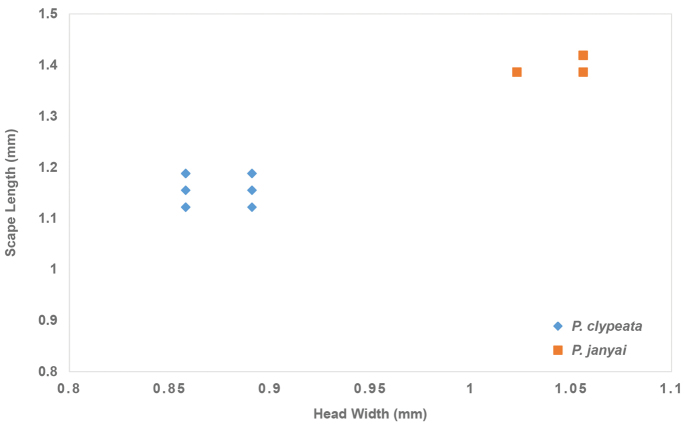
Scape length against head width in the worker.

The new species and *P. clypeata* are very similar in general appearance as they share the following characteristics: body reddish brown; frontal lobe narrow; frontal carinae closely spaced and strongly narrowed posteriorly; mandible triangular, its masticatory margin with a large apical tooth, followed by 9–10 smaller teeth, large and smaller teeth alternating; propodeal spiracle opening elliptical; in dorsal view posterior margin of petiole convex without spines. However, *P. janyai* can be easily separated from *P. clypeata* by the following characteristics: head relatively shorter (CI 72–74 in *P. janyai*; CI 61–69 in *P. clypeata*); eye clearly larger (EL 0.20 mm with eleven ommatidia on longest axis in *P. janyai*; EL 0.10 mm with five ommatidia on longest axis in *P. clypeata*); eye convex (flat in *P. clypeata*); dorsum and lateral face of head finely micropunctate without foveae (finely punctate with dense shallow foveae in *P. clypeata*); in profile view petiole clearly longer than high and in dorsal view node of petiole anteriorly as broad as posteriorly (slightly longer than high and in dorsal view node of petiole slightly narrower posteriorly in *P. clypeata*); ventral outline of petiole weakly convex (feebly concave in *P. clypeata*). Fig. 6 shows ratio of HW/SL in the workers of *P. clypeata* (37 specimens) and *P. janyai* (6 specimens) from throughout their distribution ranges; no overlapping is observed in HW / SL between the species. *P. janyai* is distinctly allopatric with *P. clypeata* in distribution. It occurs in Malay Peninsula (S Thailand and W Malaysia). On the other hand, *P. clypeata* is recorded from Sri Lanka, Vietnam, Laos, and east Thailand (Brown 1975).

Xu and Zeng (2000) identified a worker from China as *P. clypeata*. It has a body size much larger than the holotype (alate queen) of *P. clypeata* (HW 1.20 mm in the Chinese specimen; HW 1.00 mm in the holotype). In general, queens in this genus are slightly larger than workers, with corresponding modifications of thoracic sclerites (Ito 1994, Schmidt and Shattuck 2014). Thus, the Chinese specimen should be re-identified.

**Figure 7.**
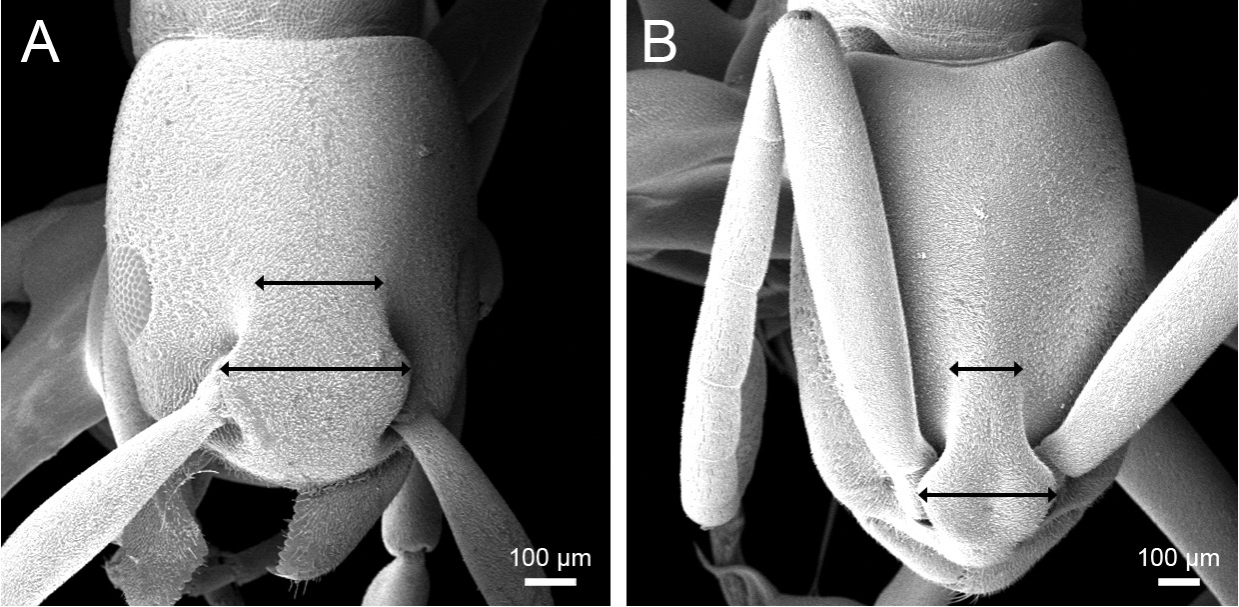
Frontal view focusing on the frontal carinae. **A** Frontal carinae very widely spaced **B** Frontal carinae relatively narrowly separated.

**Figure 8.**
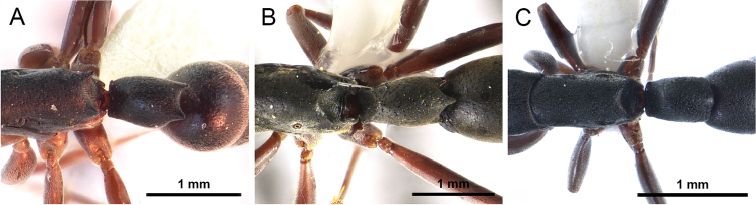
Dorsal view focusing on the petiole. **A** Posterior margin of petiole with two spines, teeth or blunt angles **B** Posterior margin of petiole with three spines, teeth or blunt angles **C** Posterior margin of petiole without distinct spines, teeth or blunt angles.

**Figure 9.**
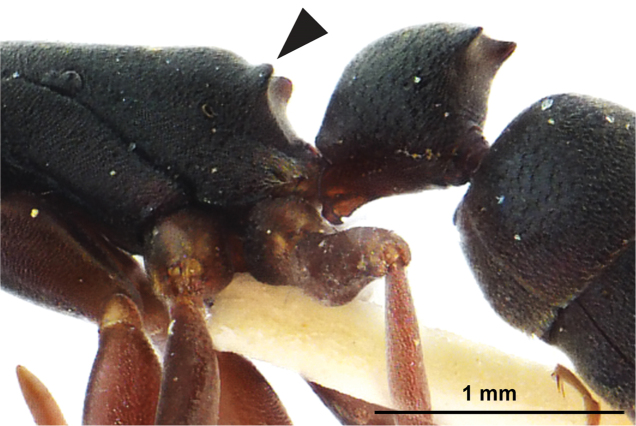
Profile view focusing on the propodeal junction of *P. quadridenta* from Thailand.

## Supplementary Material

XML Treatment for Platythyrea janyai

XML Treatment for Platythyrea clypeata

## Supplementary materials

Supplementary material 1 Data label of holotype of *Platythyrea janyai* sp.n. Data type: Data label. Explanation note: Close up of the label of Holotype of *Platythyrea janyai* sp.n. This is an open access article distributed under the terms of the Creative Commons Attribution License (CC BY 4.0), which permits unrestricted use, distribution, and reproduction in any medium, provided the original author and source are credited.Natthaporn Phengsi, Weeyawat Jaitrong, Jiraporn Ruangsittichai, Salinee Khachonpisitsak
