# Supplementary figures and images for: A sibling species of Platythyrea clypeata Forel, 1911 in southeast Asia (Hymenoptera, Formicidae, Ponerinae)
Source: Zookeys. 2018 Jan 16;(729):87–102. doi: 10.3897/zookeys.729.21378 (PMC5799728; doi:10.3897/zookeys.729.21378)

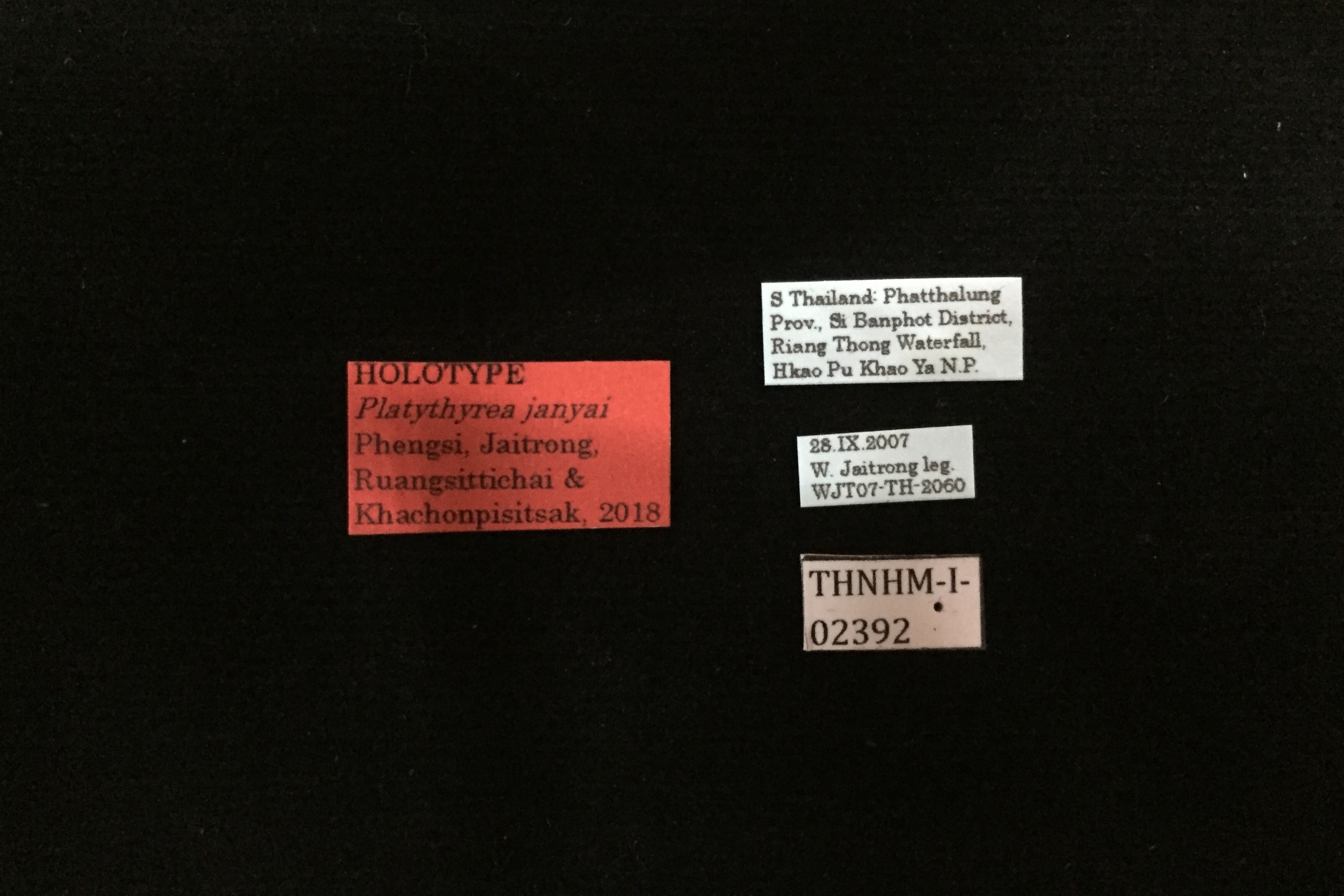

Supplement: Supplementary material 1 — Data label of holotype of Platythyrea janyai sp.n. [file zookeys-729-087-s001.jpg]
